# Parameter Estimation of Ion Current Formulations Requires Hybrid Optimization Approach to Be Both Accurate and Reliable

**DOI:** 10.3389/fbioe.2015.00209

**Published:** 2016-01-13

**Authors:** Axel Loewe, Mathias Wilhelms, Jochen Schmid, Mathias J. Krause, Fathima Fischer, Dierk Thomas, Eberhard P. Scholz, Olaf Dössel, Gunnar Seemann

**Affiliations:** ^1^Institute of Biomedical Engineering, Karlsruhe Institute of Technology, Karlsruhe, Germany; ^2^Institute of Applied and Numerical Mathematics, Karlsruhe Institute of Technology, Karlsruhe, Germany; ^3^Department of Internal Medicine III, University Hospital Heidelberg, Heidelberg, Germany

**Keywords:** electrophysiology, ionic currents, parameter estimation, particle swarm optimization, hybrid optimization, patch clamp

## Abstract

Computational models of cardiac electrophysiology provided insights into arrhythmogenesis and paved the way toward tailored therapies in the last years. To fully leverage *in silico* models in future research, these models need to be adapted to reflect pathologies, genetic alterations, or pharmacological effects, however. A common approach is to leave the structure of established models unaltered and estimate the values of a set of parameters. Today’s high-throughput patch clamp data acquisition methods require robust, unsupervised algorithms that estimate parameters both accurately and reliably. In this work, two classes of optimization approaches are evaluated: gradient-based trust-region-reflective and derivative-free particle swarm algorithms. Using synthetic input data and different ion current formulations from the Courtemanche et al. electrophysiological model of human atrial myocytes, we show that neither of the two schemes alone succeeds to meet all requirements. Sequential combination of the two algorithms did improve the performance to some extent but not satisfactorily. Thus, we propose a novel hybrid approach coupling the two algorithms in each iteration. This hybrid approach yielded very accurate estimates with minimal dependency on the initial guess using synthetic input data for which a ground truth parameter set exists. When applied to measured data, the hybrid approach yielded the best fit, again with minimal variation. Using the proposed algorithm, a single run is sufficient to estimate the parameters. The degree of superiority over the other investigated algorithms in terms of accuracy and robustness depended on the type of current. In contrast to the non-hybrid approaches, the proposed method proved to be optimal for data of arbitrary signal to noise ratio. The hybrid algorithm proposed in this work provides an important tool to integrate experimental data into computational models both accurately and robustly allowing to assess the often non-intuitive consequences of ion channel-level changes on higher levels of integration.

## 1 Introduction

The work of Hodgkin and Huxley (1952) and Beeler and Reuter (1970) are the basis of most models of cardiac electrophysiology. Coupled non-linear ordinary differential equations (ODEs) are used in such models to compute the various incorporated ionic currents. While models became more sophisticated regarding the complexity of the formulations and the data on which they are based, they describe particular types of cells from specific species and/or regions of the heart, e.g., human atrial myocytes. One longstanding model of human atrial electrophysiology was published by Courtemanche et al. (1998). As most models, it offers a description of myocytes under physiologic conditions. The investigation of other conditions, e.g., the impact of drugs, pathologies, or genetic defects on ion channels, is the target of current research.

Voltage and patch clamp measurements allow to assess ion channel kinetics, i.e., activation, deactivation, and inactivation of the channels. These methods are based on recording ion currents caused by impressed voltage protocols using electrodes inserted into the cell. The measured currents are proportional to the open probability of the channels. According to the gating concept, these data reflect altered channel kinetics and need to be integrated into models of cardiac electrophysiology in order to assess their effects on different levels of integration, e.g., their effect on the cellular, tissue, and organ level (Loewe et al., 2014b). These multi-scale simulations are often insightful and imperative for a comprehensive assessment because the altered fundamental biophysical properties enter the system in a complex and mostly non-linear way, often resulting in non-intuitive changes on higher levels of integration. A common approach is to leave the structure of the current formulation untouched and adapt the parameters used in the equations. This parameter estimation aims to minimize the difference between measured and simulated ion currents, which can be a computationally expensive and thus time-consuming process depending on the number of parameters and the abundance of measurement data. Particularly the highly non-linear, high-dimensional, and often non-convex nature of the minimization problem renders this a challenging task.

Moreover, the advent of high-throughput automated patch clamping (Dunlop et al., 2008), today being used in expression systems and manual methods on primary cardiomyocytes, lead to an increasing amount of experimental data. Therefore, computationally efficient, accurate, and most importantly robust and reliable methods for parameter estimation have to be used to integrate measurement data describing altered ion channel properties into models of cardiac electrophysiology. Often, experimental data are available on very low levels of integration (e.g., ion currents) and very high levels of integration (e.g., the ECG) with missing links on intermediate levels. Model-based approaches can bridge this gap arising from a lack of data, thus foster our understanding and facilitate the development of tailored therapeutic approaches.

Previous studies suggested different algorithms to adjust parameters of ion current formulations or cell models to reproduce measured currents, action potentials, or restitution curves (Hui et al., 2007; Bueno-Orovio et al., 2008). Besides classical gradient-based optimization, derivative-free optimization algorithms or the combination of different methods were used: e.g., particle swarm optimization (PSO) in Seemann et al. (2008) and Chen et al. (2012) and a genetic algorithm in Syed et al. (2005), Szekely et al. (2011), and Bot et al. (2012). A third class of parameter estimation algorithms is based on regression (Sobie, 2009; Sarkar and Sobie, 2010; Tøndel et al., 2014). In this paper, we evaluate two different approaches to estimate ion current formulation parameters: gradient-based trust-region-reflective (TRR) optimization (Coleman and Li, 1996) and PSO (Kennedy and Eberhart, 1995). By using synthetic as well as measured current data, we identify shortcomings of each of the algorithms when being applied to different cardiac ion currents. Thus, we propose and evaluate a novel hybrid approach coupling PSO and TRR algorithms in order to estimate the parameters accurately and reliably, i.e., being only minimally dependent on the initial guess. While hybrid optimization strategies are well known in general [see, e.g., Fan et al. (2006) and Blum and Roli (2008)], this work is the first to the best of our knowledge suggesting to combine the benefits of metaheuristic population-based algorithms (e.g., PSO) with gradient-based approaches (e.g., TRR) in the field of electrophysiology.

## 2 Materials and Methods

### 2.1 Ion Current Formulations

In this study, we chose three Hodgkin–Huxley-type ion current formulations from the Courtemanche et al. (1998) model of human trial electrophysiology. This cell model has already been applied in many simulation studies, turned out to be robust and is widely used (Wilhelms et al., 2013). The parameters of the rapid delayed rectifier potassium current *I_Kr_*, the ultra-rapid delayed rectifier potassium current *I_Kr_*, and the slow delayed rectifier potassium current *I_Ks_* were estimated. Nevertheless, other current formulations could be adapted in a similar fashion using different voltage or patch clamp data. The current formulations according to Courtemanche et al. (1998) were implemented in *Matlab (R2015a, The MathWorks, Natick, MA, USA)*. As an example, the original *I_Kr_* formulation by Courtemanche et al. (1998) is

(1) $$I_{\mathrm{Kr}}=\frac{g_{\mathrm{Kr}}x_{r}\left(V_{m}-E_{K}\right)}{1+\exp\left(\frac{V_{m}+15}{22.4}\right)},$$

where *g_Kr_* is the maximal conductance, *x_r_* the activation gating variable, *V_m_* the transmembrane voltage, and *E_K_* the potassium Nernst voltage. The gating variable *x_r_* is defined by the following ODE:

(2) $$\frac{dx_{r}}{\mathrm{dt}}=\frac{x_{r\infty }-x_{r}}{\tau_{x_{r}}},$$

with *x_r_*_∞_ being the steady-state value and $\tau_{x_{r}}$ the time constant of the gating variable *x_r_*. These two parameters are themselves dependent on *V_m_*:

(3) $$x_{r\infty }=\frac{1}{1+\exp\left(-\frac{V_{m}+14.1}{6.5}\right)},$$

(4) $$\tau_{x_{r}}=\frac{1}{\alpha_{x_{r}}+\beta_{x_{r}}}.$$

The rate constants $\alpha_{x_{r}}$ and $\beta_{x_{r}}$ are defined as a function of *V_m_* as well:

(5) $$\alpha_{x_{r}}=0.0003\frac{V_{m}+14.1}{1-\exp\left(-\frac{V_{m}+14.1}{5}\right)},$$

(6) $$\beta_{x_{r}}=7.3898\times 10^{-5}\frac{V_{m}-3.3328}{\exp\left(\frac{V_{m}+3.3328}{5.1237}\right)-1}.$$

For the *I_Kr_* formulation, 12 parameters were estimated. The structure of the other current formulations used in this study is similar. The complete set of equations necessary for the computation of all three currents *I_Kr_*, *I_Ks_*, and *I_Kur_*, including the respective parameters to be estimated, is given in Supplementary Material.

In general, ODEs, e.g., equation (2), are approximated numerically in computational cardiology since *τ_xr_* and *x_r_*_∞_ are voltage-dependent and therefore change during the cardiac cycle. However, during classic voltage clamp experiments, *V_m_* is a piecewise constant step function. Consequently, also *τ_xr_* and *x_r_*_∞_are piecewise constant functions. Thus, equation (2) can be solved analytically as suggested by Rush and Larsen (1978):

(7) $$x_{r}\left(t-t_{0}\right)=x_{r\infty }+(x_{r0}-x_{r\infty })\;\exp\left(-\frac{t-t_{0}}{\tau_{\mathrm{xr}}}\right),$$

where *t*_0_ is the time of a voltage step and *x_r_*_0_ is the corresponding initial value.

### 2.2 Voltage Clamp Data

**Figure 1:**
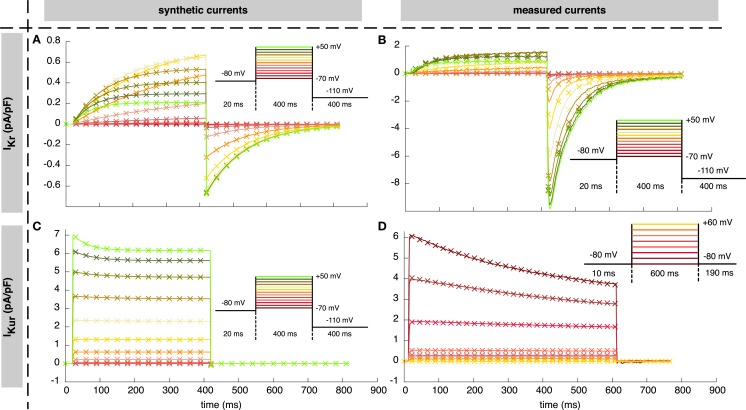
**Resulting currents using the estimated parameters**. Solid lines indicate synthetic **(A,C)** and measured **(B,D)** currents used for parameter estimation. Crosses represent the best fit obtained using the “*high*” setup of the *hybrid (PSO* + *TRR)* + *TRR* approach (every 15th sample is shown). **(A,B)** show *I_Kr_*, **(C,D)** show *I_Kur_* together with the corresponding voltage protocols.

To evaluate the accuracy and robustness of the proposed parameter estimation algorithms, both synthetic and measured current data were used. In the first step, synthetic data were generated using the standard (Courtemanche et al., 1998) parameters. We focused on two currents identified as easy to fit (*I_Kr_*) and very hard to fit (*I_Kur_*) in a pilot study. For both currents, a voltage protocol composed of 20 ms at −80 mV resting voltage, 400 ms at 13 different step voltages ranging from −70 mV to +50 mV in steps of 10 mV, and 400 ms at −110 mV resulting in a total length of 10.66 s (see Figures 1A–C) was used. Samples were taken every 2 ms. The parameter estimation algorithm was blinded to the parameter set that was used to generate these data. Using synthetic data gave the unique opportunity to gain insight into the robustness of the proposed approach. The accuracy could be evaluated as it was known that a parameter set yielding exactly the input data exists. Moreover, the identifiability of parameters could be assessed as the ground truth values were known. To assess the sensitivity to noise, the non-noisy synthetic *I_Kr_* data were duplicated 4 times and corrupted with additive Gaussian white noise yielding a signal to noise ratio (SNR) of 10, 20, 35, and 60 dB.

Additional challenges are posed when using data from real-world measurements. These data are noisy and even the most detailed current formulations do not capture every aspect of the actual biophysical entity being measured. Thus, wet-lab *I_Kr_*, *I_Kur_*, and *I_Ks_* experimental data were used in addition to the synthetic data to assess the performance under more realistic conditions. The details of the data acquisition procedures are given in Supplementary Material. The investigation conformed to the “Guide for the Care and Use of Laboratory Animals” published by the US National Institutes of Health (NIH publication No 85-23, revised 1996). The experiments were approved by the regional administrative council (Regierungspräsidium Karlsruhe, Karlsruhe, Germany) (application number G-221/12). The measured traces were sampled every 1.5–5 ms.

### 2.3 Optimization Algorithms

In this study, two kinds of optimization algorithms for the integration of voltage clamp measurement data into models of cardiac ion channels were used separately and in combination: the gradient-based TRR algorithm and the derivative-free, population-based PSO. The sum of squared errors was used as the cost function to be minimized:

(8) $$\underset{\vec{p}}{\min}\;\left({\sum_{\text{j}}\sum_{\text{i}}\left(I\left(t_{\text{i}},V_{\text{j}}\left(t_{\text{i}}\right),\vec{p}\right)-I^{*}\left(t_{\text{i}},V_{\text{j}}\left(t_{\text{i}}\right)\right)\right)}^{2}\right)$$

where *I* is the output of the ion current model depending on the vector of adjustable parameters $\vec{p}$ aiming to match the measured current I*, *t*_i_ is a discrete time, and *V*_j_ the transmembrane voltage trace, which is described by a piecewise constant function for each step voltage.

Two sets of parameter search spaces were defined. For the narrower one, parameters that enter into the equation as a summand were allowed to vary in an additive way between −60 and +60 of their value from the original Courtemanche’s formulation. An example for additive parameters is half-activation voltages. The range of parameters entering as a factor (e.g., maximum conductances) was between 0.1 and 10 times their original value. For the wider search space, the ranges were ±120 and 0.01 … 100×, respectively. The classification of parameters into additive and multiplicative parameters together with the corresponding ranges is given in Table S1 in Supplementary Material. All experiments were performed 25 times with random initial values for statistical analysis. The *Matlab* code is available from the authors on request.

#### 2.3.1 Trust-Region-Reflective

The TRR optimization algorithm is implemented in the *Matlab* function *lsqnonlin*. In brief, the concept is to approximate the function to be minimized $f\left(\vec{p}\right)$ quadratically instead of directly minimizing it. For this purpose, only the first two terms of the Taylor series of the function are considered within a so-called region of trust *r*_Δ_ around the current parameter vector ${\vec{p}}_{i}$.

(9) $$q_{i}\left(\vec{p}\right)=\;f\left({\vec{p}}_{i}\right)+\;\nabla f{\left({\vec{p}}_{i}\right)}^{T}\left(\vec{p}-{\vec{p}}_{i}\right)+\;\frac{1}{2}{\left(\vec{p}-{\vec{p}}_{i}\right)}^{T}\nabla^{2}f\left({\vec{p}}_{i}\right)\left(\vec{p}-{\vec{p}}_{i}\right),$$

(10) $$\underset{||s||\leq r_{\Delta }}{\min}\;q_{i}\left({\vec{p}}_{i}+\vec{s}\right).$$

The region of trust is adjusted in the course of the optimization depending on the quality of the second order approximation. The more accurate the approximation was in the current iteration, the larger *r*_Δ_ will be in the next iteration. As stopping criteria of this iterative algorithm, the minimum change of the function value (*fTol*) and the minimum change of the norm of the parameter vector $\vec{p}$ (*pTol*) were set to 1 × 10^−11^ (pA/pF)^2^ in this work. The maximum number of function evaluations (*maxFunEval*) was 5 × 10^5^ and the maximum number of iterations (*maxIter*) 1 × 10^5^.

#### 2.3.2 Particle Swarm Optimization

In Wilhelms et al. (2012), the TRR algorithm alone was sensitive to the choice of the initial parameter vector, resulting in estimates corresponding to different local minima of the cost function. Therefore, a derivative-free algorithm was implemented additionally in this study. The PSO algorithm is based on, as the name implies, swarming theory describing the behavior of, e.g., flocking birds (Kennedy and Eberhart, 1995). In general, the movement of a population of particles starting from random initial positions within a parameter space searching for a globally best solution is described mathematically. For this purpose, the velocity ${\vec{v}}_{i}$ and position ${\vec{p}}_{i}$ of each “particle” were computed in each iteration. The globally best position found so far by the entire swarm $\left({\vec{b}}_{g}\right)$ and its own best position found so far $\left({\vec{b}}_{i}\right)$ were known to each particle as shown, e.g., in Poli et al. (2007):

(11) $${\vec{v}}_{i}\leftarrow \chi \left({\vec{v}}_{i}+\vec{U}\left(0,\phi_{1}\right)⊗\left({\vec{b}}_{i}-{\vec{p}}_{i}\right)+\vec{U}\left(0,\phi_{2}\right)⊗\left({\vec{b}}_{g}-{\vec{p}}_{i}\right)\right),$$

(12) $${\vec{p}}_{i}\leftarrow {\vec{p}}_{i}+{\vec{v}}_{i},$$

where $\vec{U}\left(0,\phi_{1}\right)$ and $\vec{U}\left(0,\phi_{2}\right)$ are vectors of the same length as $\vec{p}$ of uniformly distributed random numbers (between 0 and *ϕ*_1_ or *ϕ*_2_) and ⊗ is a component-wise multiplication. Clerc and Kennedy (2002) chose *ϕ*_1_ = *ϕ*_2_ = 2.05 and defined the constriction coefficient χ as

(13) $$\chi =\frac{2}{\phi -2+\sqrt{\phi^{2}-4\phi }}\approx 0.73,$$

with *ϕ* = *ϕ*_1_ + *ϕ*_2_. The number of iterations *L* was set to 1 × 10^3^. If the iteratively computed parameter values were out of the defined boundaries, they were set to a random value within 25% of the parameter range starting from the boundary which was crossed. Consequently, the entire swarm was supposed to move toward the globally best solution within the defined parameter ranges. The computation of the cost function for each particle in an iteration was parallelized in *Matlab*. The number of particles *N* was varied between 24 and 12,288 with their number being doubled from one setup to the next. As none of the algorithms performed satisfactory (see Results), the two algorithms were combined to leverage their individual advantages.

#### 2.3.3 Combination of Algorithms

**Figure 2:**
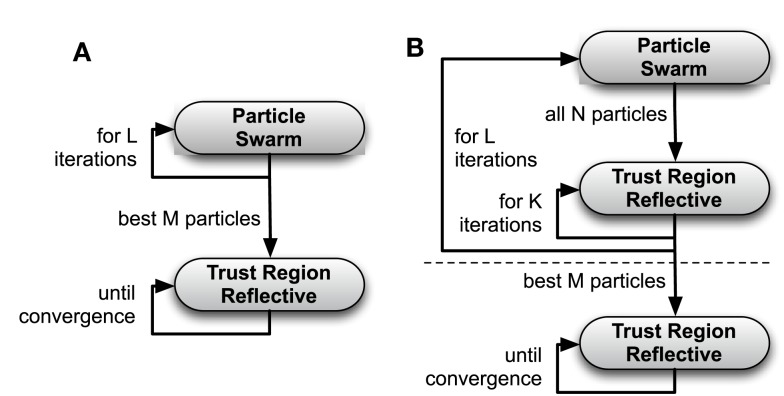
**Flow chart of the *two-stage PSO* + *TRR* algorithm (A) and the *hybrid (PSO* + *TRR)* + *TRR* algorithm (B)**. The steps above the horizontal, dashed line in **(B)** are referred to as *hybrid (PSO* + *TRR)*.

As TRR alone was prone to get stuck in local minima depending on the choice of the initial parameter vector, a straight-forward two-stage approach was chosen such that the derivative-free PSO was used for the selection of initial parameter vectors for TRR (referred to as “*two-stage PSO* + *TRR*”). The best *M* = 12 parameter sets found using PSO were passed on as initial estimates for subsequent TRR optimization (see Figure 2A). The number of particles *N* was varied between 24 and 12,288 as for pure PSO.

Because also this two-stage approach did not perform satisfactorily in all scenarios, a hybridization strategy [referred to as “*hybrid (PSO* + *TRR)*”] was implemented combining gradient-based and derivative-free optimization in each of the PSO iterations as detailed in Algorithm 1. All *N* particles with their current parameter vectors ${\vec{x}}_{i}$ underwent a fixed number of *K* TRR iterations in each PSO iteration. Thus, ${\vec{v}}_{i}$ was modified by TRR in each iteration. Optionally, TRR was run until convergence for the best *M* = 12 particles at the end [referred to as “*hybrid (PSO* + *TRR)* + *TRR*”]. Figure 2B illustrates the hybrid strategy for which three setups were evaluated: “*low*” (K = 5, *L* = 250, *N* = 96), “*medium*” (*K* = 10, *L* = 500, *N* = 192), and “*high*” (*K* = 20, *L* = 1000, *N* = 384).

**Algorithm 1:** **“hybrid (PSO + TRR) + TRR” optimization approach**.

**for** *itPSO* < *L* **do**
**for** *i* < *N* **do**
${\vec{v}}_{i}\leftarrow \chi \left({\vec{v}}_{i}+\vec{U}\left(0,\phi_{1}\right)⊗\left({\vec{b}}_{i}-{\vec{p}}_{i}\right)+\vec{U}\left(0,\phi_{2}\right)⊗\left({\vec{b}}_{g}-{\vec{p}}_{i}\right)\right)$
${\vec{\tilde{p}}}_{i}\leftarrow {\vec{p}}_{i}+{\vec{v}}_{i}$
enforce boundary constraints on ${\vec{\tilde{p}}}_{i}$
**for** *itTRR* < *K* **do**
perform TRR iteration on ${\vec{\tilde{p}}}_{i}$
**end for**
${\vec{v}}_{i}\leftarrow {\vec{\tilde{p}}}_{i}-{\vec{p}}_{i}$
${\vec{p}}_{i}\leftarrow {\vec{p}}_{i}+{\vec{v}}_{i}$
**end for**
**end for**
sort $\vec{b}[]$ by ascending squared error
**for** *i* < *M* **do**
**while** (not converged) ∧ (*itTRR* < *maxTRR*) **do**
perform TRR iteration on ${\vec{b}}_{i}$
**end while**
**end for**

## 3 Results

### 3.1 Estimation Using Synthetic Input Data

The different combinations of algorithms were tested on synthetic data in a first step. For these data, a parameter set yielding exactly the input data (i.e., an error of zero) exists and was available for comparison.

#### 3.1.1 One-Stage Approach

**Figure 3:**
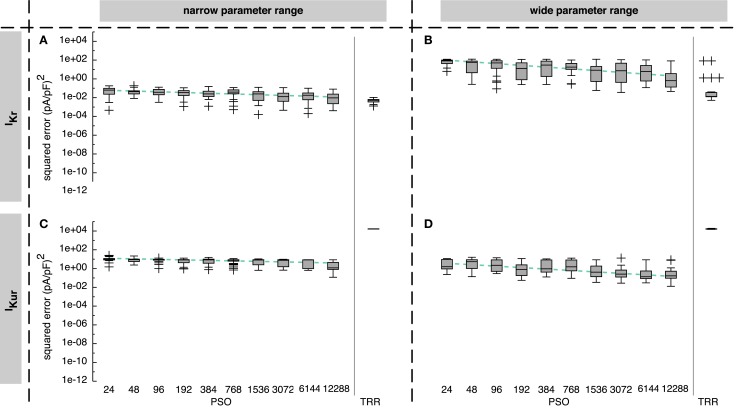
**Sum of squared errors achieved by pure particle swarm optimization (PSO) and pure trust region-reflective (TRR) optimization for synthetic *I_Kr_* (A,B) and *I_Kur_* (C,D) data**. The number of particles N for PSO was varied. In **(A,C)**, narrow parameter ranges were used, whereas in **(B,D)**, the search space was wider. Box plots represent 25 experiments each; the dashed line indicates a linear regression of the median values in the graph coordinate system.

Comparing the two one-stage approaches, TRR performed best for *I_Kr_*, whereas PSO yielded better results by four orders of magnitude for *I_Kur_* (see Figure 3).

For the *I_Kr_* formulation and the narrow parameter range, pure PSO yielded squared errors between 1 × 10^−4^ and 0.18 (pA/pF)^2^ with a tendency toward lower errors for a higher number of particles *N* [median error 6.4 × 10^−2^ (pA/pF)^2^ for *N* = 24 and 9.5 × 10^−3^ (pA/pF)^2^ for *N* = 12,288]. Pure TRR optimization with random start vectors yielded a lower median error of 5.1 × 10^−3^ (pA/pF)^2^ and a smaller spread compared to pure PSO (see Figure 3A). Using the wide parameter range, the error for pure PSO was higher by about 3 orders of magnitude (see Figure 3B). While the median error for pure TRR optimization was unaffected by widening the parameter range, five of the experiments yielded significantly higher errors (Figure 3B). Among the termination criteria introduced in Section 1, the decisive criterion for TRR was the tolerance of the change of the norm of $\vec{p}$ (*pTol*) for all experiments using the narrow range. For the wide range, *pTol* was decisive in 19% of the cases, whereas the remaining runs were terminated due to the tolerance of the norm of the squared errors (*fTol*).

Looking at *I_Kur_* results, two differences were striking: first, pure TRR optimization performed significantly worse compared to pure PSO by more than 4 orders of magnitude (see Figure 3C). Second, the lower squared error obtained by the wide parameter range compared to the narrow range (see Figure 3D). Pure PSO was better by 80% (*N* = 12,288) using the wide range, pure TRR was better by 12%. TRR was terminated due to *pTol* in 3% of the cases, due to *fTol* in 66%, and due to the maximum number of iterations (*maxIter*) in 31% for the narrow range. For the wide range, *pTol* was decisive in 10% of the cases, *fTol* in 50%, and *maxIter* in 40%.

**Figure 4:**
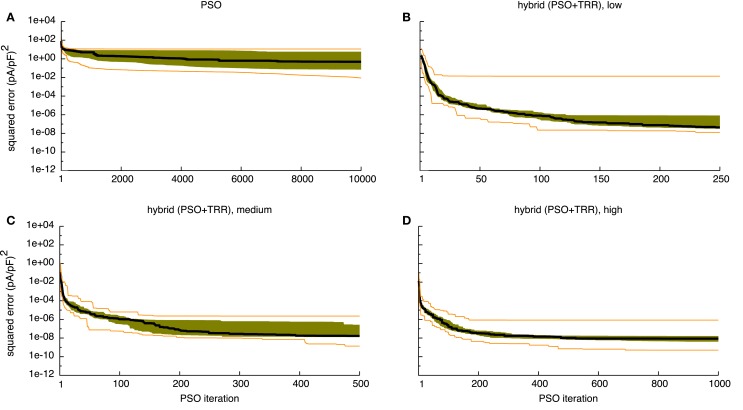
**Sum of squared errors convergence behavior of pure PSO (A) and *hybrid (PSO* + *TRR)* (B–D) for synthetic *I_Kur_* data**. 25 experiments were performed, the black line indicates the median, the orange lines the minimum and the maximum, the green area covers the two central quartiles. The number of particles *N*, the number of PSO iterations *L*, and the number of inner TRR iterations *K* was increased from “*low*” **(B)** via “*medium*” **(C)** to “*high*” **(D)**.

Figure 4A demonstrates that the first half of the PSO iterations yielded most of the change for *I_Kur_*. While the median error dropped until around 8,500 iterations for *I_Kr_* (see Figure S1A in Supplementary Material), the maximum error remained almost unchanged after the first few iterations for both currents.

#### 3.1.2 Two-Stage PSO + TRR Approach

**Figure 5:**
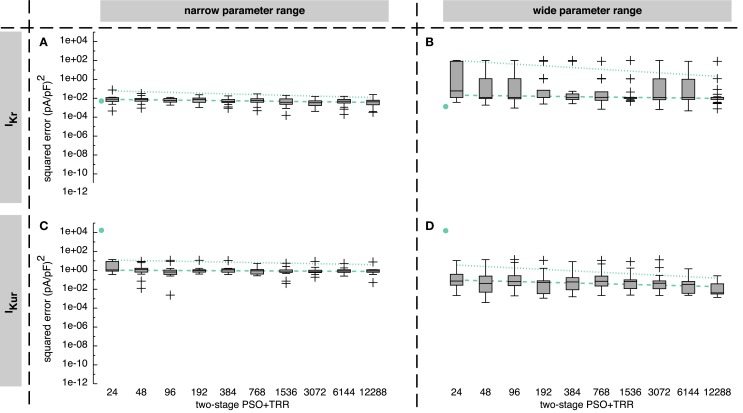
**Sum of squared errors achieved by *two-stage PSO* + *TRR* optimization for synthetic *I_Kr_* (A,B) and *I_Kur_* (C,D) data**. The number of particles *N* was varied. In **(A,C)**, narrow parameter ranges were used, whereas in **(B,D)**, the search space was wider. Box plots represent 25 experiments each. The dashed line indicates a linear regression of the median values in the graph coordinate system. The dot on the left of each panel represents the median of pure TRR optimization; the dotted line represents a linear regression of the median values of pure PSO (compare Figure 3).

Combining PSO and TRR by passing the best *M* = 12 particles on to subsequent TRR optimization as start vectors reduced the squared error compared to pure PSO in all cases and compared to pure TRR in most cases (see Figure 5). The results for *I_Kur_* were improved by sequential combination of PSO and TRR to a larger extent than those for *I_Kr_*. The improvement was larger for the wide parameter range than for the narrow one.

Using the narrow parameter range, the reduction of *I_Kr_* squared error was 87% for *N* = 24 and 56% for *N* = 12,288 (see Figure 5A). Thus, the median error of the two-stage approach for *N*  ≥ 1536 was smaller than for any single algorithm. However, the worst estimate obtained by pure TRR was better than the worst estimate obtained by *two-stage (PSO* + *TRR)*. Using the wide *I_Kr_* parameter range increased the spread of resulting squared errors for *two-stage (PSO* + *TRR)* accompanied by a slight increase of the median error [1.2 × 10^−2^ (pA/pF)^2^ compared to 4.2 × 10^−3^ (pA/pF)^2^, see Figure 5B]. In contrast, the median error was increased significantly accompanied by a slight increase of the spread for pure PSO (Figure 3B). The TRR step of the approach was terminated due to *pTol* in all cases for the narrow range. For the wide range, *fTol* was decisive in 16% of the cases.

Looking at *I_Kur_*, *two-stage (PSO* + *TRR)* improved the result by 6% using the narrow range (see Figure 5C) and by 88% using the wide range considering *N* = 12,288 (see Figure 5D). Widening the parameter range caused a decrease of the median error and an increase of the spread for *I_Kur_* in the *two-stage (PSO* + *TRR)* scenario as was the case for pure PSO. The crucial stopping criterion was *maxIter* in 80% of the cases for the narrow range. For the wide range, *pTol*/*maxIter*/*fTol* were decisive in 51/30/29% of the cases, respectively.

#### 3.1.3 Hybrid Approach

**Figure 6:**
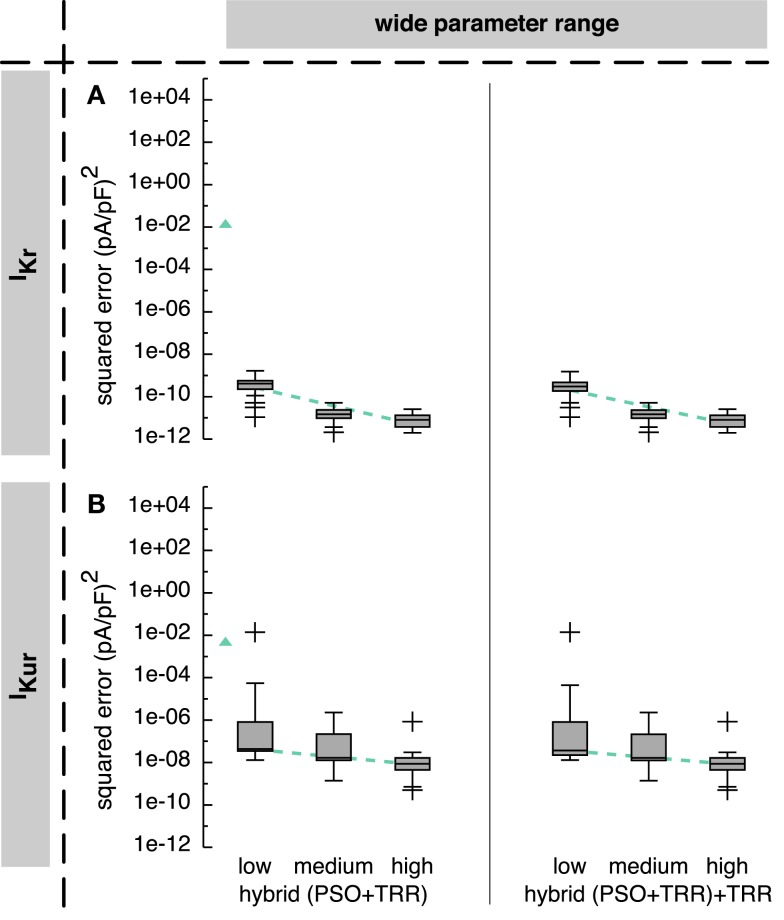
**Sum of squared errors achieved by *hybrid (PSO* + *TRR)* and *hybrid (PSO* + *TRR)* + *TRR* optimization for synthetic *I_Kr_* (A) and *I_Kur_* (B) data**. Parameter values were restricted to the wide range. The number of particles *N*, the number of PSO iterations *L*, and the number of inner TRR iterations *K* was increased from “*low*” via “*medium*” to “*high*.” Box plots represent 25 experiments each; the dashed line indicates a linear regression of the median values in the graph coordinate system. The triangle on the left of each panel represents the median of *two-stage PSO* + *TRR* for *N* = 12,288 (compare Figure 5).

The hybrid approach reduced the squared error by more than 5 orders of magnitude for both *I_Kr_* and *I_Kur_* compared to the sequential combination of both algorithms (see Figure 6).

Using the wide parameter range, the *hybrid (PSO* + *TRR)* approach yielded median squared errors smaller than 1 × 10^−9^ (pA/pF)^2^ for *I_Kr_* (see Figure 6A) and smaller than 1 × 10^−7^ (pA/pF)^2^ for *I_Kur_* (see Figure 6B), thus 7 and 5 orders of magnitude lower than the *two-stage PSO* + *TRR* approach. The “*low*” setup with the lowest number of particles, PSO iterations, and inner TRR iterations yielded a single outlier squared error of 1.4 × 10^−2^ (pA/pF)^2^ for *I_Kur_*. For this outlier, the squared error did not decrease significantly after the first 10 iterations. For the “*medium*” and “*high*” setups, the maximum errors were 5.2 × 10^−11^ (pA/pF)^2^ for *I_Kr_* and 2.3 × 10^−6^ (pA/pF)^2^ for *I_Kur_*, respectively.

Restricting the parameters to the narrow range caused only minor changes for *I_Kr_* (see Figure S2A in Supplementary Material). For *I_Kr_*, similar effects for the *hybrid (PSO* + *TRR)* approach were observed as was the case for *two-stage PSO* + *TRR*. The spread of the squared error was higher for the narrow ranges with several outliers up to 3.5 × 10^−1^ (pA/pF)^2^ using the “*medium*” setup (see Figure S2B in Supplementary Material).

The median error converged to an interval within one order of magnitude of its final value in 112/167/128 iterations for the “*low*”/“*medium*”/“*high*” *I_Kur_* setups using the wide parameter range (see Figures 4B–D). However, for the “*low*” setup, the median error was still decreasing during the final iterations. As was the case for pure PSO, convergence was slower for *I_Kr_* with 125/217/232 iterations, respectively (see Figure S1 in Supplementary Material). Running TRR until convergence in the *hybrid (PSO* + *TRR)* + *TRR* approach did reduce the squared error by <1% (see Figure 6 and Figure S2 in Supplementary Material). The decisive stopping criterion for the final TRR step was *pTol* in ≈80% of the cases and *fTol* in ≈20% with no marked differences between currents, parameter ranges, and algorithm setups.

**Figure 7:**
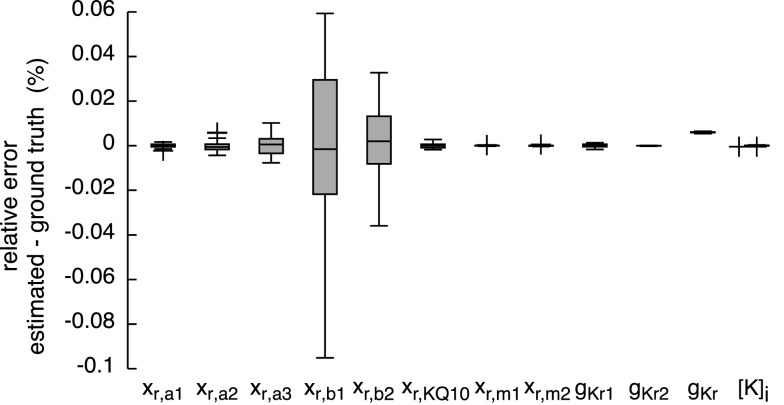
**Relative error of the estimated parameters using synthetic *I_Kr_* data and the *hybrid (PSO* + *TRR)* + *TRR* approach in the “*high*” configuration**. Parameter deviations were normalized to their ground truth values. Box plots represent 25 experiments.

**Figure 8:**
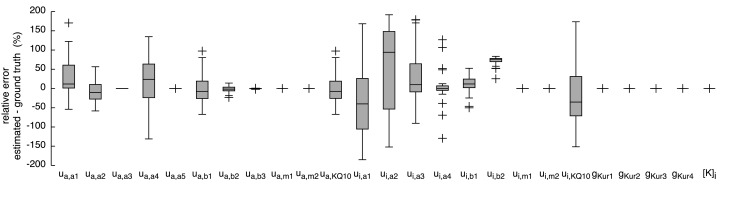
**Relative error of the estimated parameters using synthetic *I_Kur_* data and the *hybrid (PSO* + *TRR)* + *TRR* approach in the “*high*” configuration**. Parameter deviations were normalized to their ground truth values. Box plots represent 25 experiments.

The resulting currents of the *hybrid (PSO* + *TRR)* + *TRR* estimation yielding the highest squared error are shown in Figure 1A for *I_Kr_* and in Figure 1C for *I_Kur_*. The difference between the simulated currents using the estimated parameters and the synthetic input data is shown in Figures S3A,C in Supplementary Material (note the different scale compared to Figure 1). Figures 7 and 8 show the relative deviation of the estimated parameters from the ground truth values. For *I_Kr_*, all 12 parameters were estimated with an accuracy of <0.1% deviation. For *I_Kur_*, several of the 25 estimated parameters deviated significantly from the ground truth values. Likely reasons for several *I_Kur_* parameters being hardly identifiable are discussed below.

### 3.2 Influence of Noise

**Figure 9:**
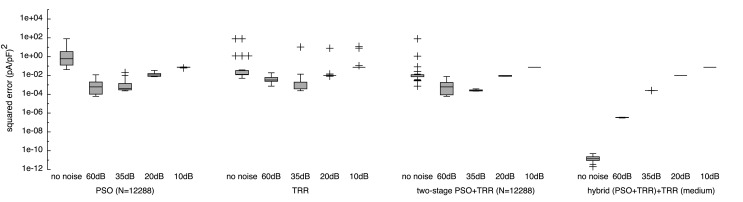
**Sensitivity of the sum of squared errors to noise in synthetic *I_Kr_* data using pure PSO, pure TRR, *two-stage (PSO* + *TRR)*, and *hybrid (PSO* + *TRR)* + *TRR* optimization**. The error was measured with respect to the original, non-noisy data. Parameters were restricted to the wide ranges. Box plots represent 25 experiments.

To assess how additive Gaussian white noise in the input data affects the performance of the algorithms, we used synthetic *I_Kr_* input data with SNRs of 10, 20, 35, and 60 dB in addition to the non-noisy data evaluated above. While the non-hybrid approaches yielded worse results when improving the SNR to values above 35 dB, the hybrid approach was able to cope with data with higher SNR as well (see Figure 9).

The cost function seen by the optimization algorithm was the sum of squared differences between the model output using the estimated parameters and the noisy input data. This metric got worse for increasing noise levels (lower SNR) for all algorithms (see Figure S4 in Supplementary Material). For a moderate noise level of 60 dB, *hybrid (PSO* + *TRR)* yielded a lower error [2.7 × 10^−4^ (pA/pF)^2^ for “*medium*”] compared to simpler approaches [8.8 × 10^−4^ (pA/pF)^2^ for PSO with *N* = 12,288]. For increased noise levels, this difference vanished [2.6 × 10^1^ (pA/pF)^2^ for all approaches using data with SNR = 10 dB]. The squared error for non-noisy input data was higher than that obtained using SNR = 60 dB data for all approaches but *hybrid (PSO* + *TRR)*. The hybrid approach yielded optimal results in all noise scenarios considering the sum of squared differences between the non-noisy and the noisy input data shown in Figure S4 in Supplementary Material as a lower boundary for the sum of squared errors. For SNR values below 35 dB, PSO and *two-stage PSO* + *TRR* yielded optimal results as well.

The squared error with respect to the non-noisy, ground truth input data rather than the noisy data, which the optimization saw in the cost function is shown in Figure 9. The main difference is that when relating the result to the ground truth, the squared error was lower. For this metric, the lower boundary is zero. Using *hybrid (PSO* + *TRR)* + *TRR* “*medium*,” the squared error for SNR = 60 dB was lower by 3 orders of magnitude when comparing to the ground truth. For pure PSO, pure TRR and *two-stage (PSO* + *TRR)*, the squared error was lower for SNR = 35 dB than for 60 dB. This was not the case for *hybrid (PSO* + *TRR)* + *TRR*, which still showed a monotonic increase of error for increasing noise levels.

### 3.3 Estimation Using Measured Input Data

**Figure 10:**
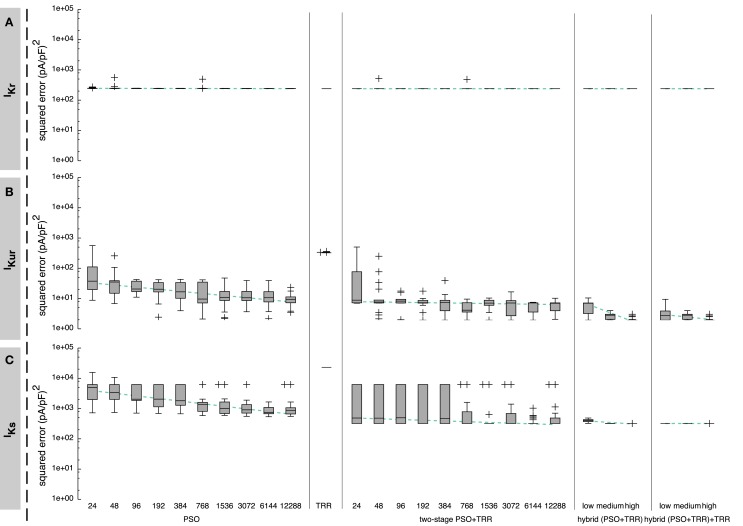
**Sum of squared errors achieved by pure PSO or TRR, *two-stage PSO* + *TRR*, *hybrid (PSO* + *TRR)*, and *hybrid (PSO* + *TRR)* + *TRR* optimization for measured *I_Kr_* (A), *I_Kur_* (B), and *I_Ks_* (C) data**. Parameter values were restricted to the wide range. Note the different scaling compared to Figures 3, 5, and 6. For the one-stage and two-stage PSO approaches, the number of particles *N* was varied. For the hybrid approaches, the number of particles *N*, the number of PSO iterations *L*, and the number of inner TRR iterations *K* was increased from “*low*” via “*medium*” to “*high*.” Box plots represent 25 experiments each; the dashed line indicates a linear regression of the median values in the graph coordinate system.

In a second step, the algorithms were tested using measured current data. For these data, a parameter vector yielding an error close to zero does normally not exist due to simplifications in the mathematical model and measurement noise. The hybrid approach always yielded the result with the lowest squared error (see Figure 10). For *I_Kr_*, results were within one order of magnitude for all investigated optimization approaches. Results were improved by 1 order of magnitude using the hybrid approach for *I_Kur_*, whereas the main advantage of the hybrid over the other approaches for *I_Ks_* was reduced variability in the results by 1 order of magnitude.

As was the case for the synthetic data, the spread of the squared error was smaller when TRR optimization was incorporated for *I_Kr_* (see Figure 10A). The current with a slow upstroke following the first voltage step was adjusted well by the model (see Figure 1B). The difference between the simulated and measured *I_Kr_* is shown in Figure S3B in Supplementary Material. The step down to −120 mV caused a fast inward current, which quickly went back to zero again. This recovery could not be adjusted well by the Courtemanche’s current formulation which uses only one gate with a time constant and one instantaneous gate (*τ* = 0 *ms*), thus forcing the same time constant/voltage relation for both step responses.

The characteristics of the results obtained using the measured *I_Kur_* were comparable to the ones using synthetic data (see Figure 10B). First, pure PSO yielded a significantly lower squared error [9.4 (pA/pF)^2^ for *N* = 12,288] compared to TRR [3.2 × 10^2^ (pA/pF)^2^]. Second, *hybrid (PSO* + *TRR)* was superior to *two-stage PSO* + *TRR* regarding both the median and the maximum error. The currents simulated using the estimated parameters are visually almost indistinguishable from the measured currents (see Figure 1D). The difference between the simulated and measured *I_Kur_* is shown in Figure S3D in Supplementary Material.

The performance of the algorithms regarding *I_Ks_* was only assessed using measured data. In general, results were comparable to *I_Kur_* with PSO yielding lower squared errors than TRR for sufficiently large *N* (see Figure 10C). Two differences were striking: First, the results varied significantly stronger for *I_Ks_* using the *two-stage PSO* + *TRR* approach compared to *I_Kur_* and compared to pure PSO. Second, the main difference between *hybrid (PSO* + *TRR)* and *two-stage PSO* + *TRR* was a reduced variance rather than a reduced median error. Figure S5B in Supplementary Material shows the fitted current together with the input data. The difference plot in Figure S5B in Supplementary Material demonstrates that the steady-state currents were fitted well. On the other hand, the biphasic nature of the response to the first voltage step composed of an almost immediate upstroke and a slower, exponential one could not be covered by the Courtemanche et al. (1998) *I_Ks_* formulation comprising four identical *x_s_* gates.

### 3.4 Computing Times

All experiments were performed on a 2.4-GHz Intel Xeon E5645 machine with 64 GB RAM under Mac OS X (*Apple Inc., Cupertino, CA, USA*). Table 1 gives the median computing times (*n* = 25) of the different algorithms. In general, PSO was the fastest algorithm. TRR was faster when being initialized with particle swarm optimized start vectors (*two-stage PSO* + *TRR*) as compared to random start vectors even when considering the time needed for PSO. This was not the case for the synthetic *I_Kr_* data for which TRR was faster than PSO by 1 order of magnitude (data not shown). *I_Kur_* was computationally more expensive with PSO (*N* = 1,536) being faster than TRR by a factor of 2.8. The *hybrid (PSO* + *TRR)* approach took the longest time (2.5 h for *I_Kr_*, 6.1 h for *I_Kur_*, and 1.0 h for *I_Ks_*). Regarding the measured data, the time needed for convergence of the final TRR step in the *hybrid (PSO* + *TRR)* + *TRR* approach was <2 s for *I_Kr_*, <1167 s for *I_Kur_* in all cases, and <700 s for *I_Ks_* in all cases. For *I_Kur_*, this was significantly longer than for synthetic data (20 s).

**Table 1:** **Median computing times (*n* = 25) for the parameter estimation using the measured data**.

	*I_Kr_* (s)	*I_Kur_* (s)	*I_Ks_* (s)
PSO	839	569	341
TRR	2763	1570	599
*Two-stage PSO* + *TRR*	854	832	361
*Hybrid (PSO* + *TRR)*	8923	21840	3687
*Hybrid (PSO* + *TRR)* + *TRR*	8925	21894	3692

*For pure PSO and *two-stage PSO* + *TRR*, the number of particles was set to *N* = 1536. For *hybrid*, the “*medium*” setup was used*.

For all approaches, more than 95% of the time was spent on the evaluation of the cost function.

## 4 Discussion

In the present study, the gradient-based TRR algorithm, the population-based PSO and different combinations of both approaches were evaluated regarding their suitability for parameter estimation of cardiac ion channel models with respect to accuracy, robustness, and reliability. Toward this end, synthetic and measured *I_Kr_*, *I_Kur_*, and *I_Ks_* data were used.

### 4.1 Algorithm Performance

The performance of each algorithm alone was highly dependent on the type of problem: TRR yielded significantly better results for *I_Kr_*, whereas PSO performed significantly better for *I_Kur_*. An observation common to both currents was that a high number of iterations did not help to prevent bad estimates if the particle swarm moved in a wrong direction during the very first iterations. Combining both algorithms in a one-after-the-other manner (*two-stage PSO* + *TRR*) reduced the median error, however at the expense of larger spread, particularly using wide ranges. Therefore, the two algorithms were coupled more tightly incorporating TRR into each PSO iteration. This *hybrid (PSO* + *TRR)* approach yielded consistently low median error values together with a robust and reliable estimation, i.e., minimal spread when starting from different initial guesses. A reason explaining these characteristics is that the cost function for *I_Kr_*, on the one hand, was relatively well fit by the quadratic approximation made by TRR and not much distorted by local minima. *I_Kur_*, on the other hand, was characterized by extensive plateaus with narrow and steep minima. Thus, the random aspect of the PSO helped to overcome the plateaus while it was necessary to consider the gradient in each iteration to identify and descend into the actual minima.

We showed that using non-hybrid approaches, the parameter estimation gets worse when the noise conditions in the experimental design are improved. By reducing noise, the margin of parameters yielding the optimal result becomes narrower. This leads to more articulated minima in the parameter space. Our explanation for the worse performance of the non-hybrid approaches for less noisy data is that they get stuck in local minima. By adding noise, the minima get blurred. Hence, the algorithms are less prone to get stuck in local minima. This finding implies that the algorithm being used for parameter estimation must be capable of handling data of the quality at hand. We could show that our hybrid approach is not limited in this respect and can be applied to data of arbitrary high quality. For noisy data, we showed that the hybrid approach yields the optimal approach up to the theoretical limit.

While the accuracy of the hybrid approach was higher than required for probably most use cases considering realistic noise conditions, the ability to generate reliable estimates in a single run is of great importance in practice. The novel hybrid approach proved to fulfill this requirement as shown particularly for measured *I_Kur_* and *I_Ks_* data (see Figure 10). The deviation of the measured data and the best fit observed for *I_Kr_* and *I_Kur_* is probably due to (i) minor differences between the model and the expression system (e.g., hERG codes only for the *α*-subunit of *I_Kr_*, oocytes vs. other expression systems, room temperature measurements), (ii) noise, (iii) model simplifications, and (iv) the averaging of measurements in several cells. Regarding (iii), the Courtemanche et al. *I_Kr_* formulation does, e.g., not fully capture human atrial *I_Kr_* as shown by Bett et al. (2011). The immediate step response observed for *I_Ks_*, which could not be reproduced by the model (see Figure S5 in Supplementary Material), might be due to background currents, which could be addressed and eliminated in pre-processing steps. Regarding (iv), it is important to keep in mind that the system is non-linear, thus the model might represent the current recorded in each single cell but not the average current.

### 4.2 Recommended Approach

Our results show that the *hybrid (PSO* + *TRR)* + *TRR* “*medium*” setup satisfies accuracy and robustness requirements while keeping the runtime below 7 h, thus striking a good balance between computational cost and quality of fit. The data suggest that the number of iterations could be reduced without a significant loss of quality. Final TRR optimization until convergence did not improve the result significantly. For the synthetic data, it did also account for only a very minor share of computation time as the algorithm had already almost converged. Using measured data, final TRR took up to 35× longer for *I_Kur_* indicating no well-defined, convex minimum. Interestingly, this difference in final TRR runtime between synthetic and measured data could not be observed for *I_Kr_*, again indicating the benign nature of this optimization problem. The minimal spread of the resulting squared error using the *hybrid (PSO* + *TRR)* + *TRR* “*medium*” for all currents (see Figure 10) shows that a single run is sufficient. This advantage outweighs the additional computation time, which was longer by one order of magnitude for the hybrid approach as compared to sequential optimization. For the *two-stage PSO* + *TRR* optimization, several passes with different start vectors are needed to reliably get close to the minimum within one order of magnitude. The pronounced parallel nature of the problem stemming from the fact that the cost function can be evaluated separately for each “particle” could be exploited by leveraging highly parallel hardware, such as GPUs reducing the computation time further.

When using *two-stage PSO* + *TRR*, parameter ranges should be set neither too narrow (compare Figure 5C and Figure 5D) nor too wide (compare Figure 5A and Figure 5B). The effect of lower errors using a wider parameter range observed for *I_Kur_* might be due to the way the boundaries are enforced in our variant of the PSO algorithm: If a parameter leaves the predefined search space, it is placed randomly within a 25% margin. However, narrowing the 25% range with iterations did not alter results significantly (data not shown). On the other hand, the *hybrid (PSO* + *TRR)* approach did not show significant sensitivity to the width of the parameter range advocating the use of this algorithm. In conclusion, our newly proposed hybrid approach yields accurate and reliable estimates for both measured and synthetic input data within few hours.

While metaheuristic approaches as PSO (Seemann et al., 2008; Chen et al., 2012) or genetic algorithms (Syed et al., 2005; Szekely et al., 2011; Bot et al., 2012) have been proposed earlier, we are the first to combine the two approaches in a hybrid scheme (Fan et al., 2006; Blum and Roli, 2008) in the field of cardiac electrophysiology. Our results show that such hybridization is imperative to obtain both accurate and reliable estimates. Preliminary experiments using a genetic algorithm suggested performance comparable to pure PSO (data not shown).

### 4.3 Limitations

A limitation of the estimation pipeline is that the system being fitted is not necessarily in steady state as only one stimulus is applied during each evaluation of the cost function. However, particularly during later iterations, parameters change rather slightly, thus causing only minor transient oscillations. Moreover, non-steady-state artifacts are less of a problem in single current formulations as compared to whole-cell models, e.g., when fitting current densities (Loewe et al., 2014a). Hence, we identified the presented approach as a good balance between runtime and steady-state approximation.

Parameter estimation algorithms based on regression (Sobie, 2009; Sarkar and Sobie, 2010; Tøndel et al., 2014) can in part provide information on sensitivity and parameter identifiability besides an estimate of the parameters. This information cannot be obtained using our approach on experimental data. However, we anticipate that regression-based approaches will also struggle with challenging formulations, e.g., *I_Kur_*. Considering that some of the *I_Kur_* parameters were hardly identifiable by the voltage protocol used in our study, a combination with approaches like proposed in Tøndel et al. (2014) or Fink and Noble (2009) to evaluate parameter identifiability appears advisable. In the first step, the set of parameters to be estimated would be identified; then, our hybrid approach could be used to actually estimate the value of these parameters.

In this study, we evaluated the performance of parameter estimation algorithms using synthetic and measured data of three different potassium currents covering a range of characteristics (e.g., fast *I_Kur_* kinetics vs. rather slow *I_Kr_*) and based on the availability of experimental data. Nevertheless, the results should also be applicable to other currents carried by potassium or other ions. Even faster kinetics, e.g., for *I_Na_* will not be a problem as long as the currents are measured with sufficient temporal resolution as the algorithm itself is time-agnostic. The model of Courtemanche et al. (1998) was used in this work since it has already been applied in numerous simulation studies of, e.g., atrial fibrillation and convinced in a benchmark of different human atrial models (Wilhelms et al., 2013). Nevertheless, the presented method can be applied to models of other types of cells [e.g., a ventricular model such as ten Tusscher et al. (2004)] or Markov models of ion channels rather than pure Hodgkin–Huxley-type models. The results found in this study should hold for these kinds of models, as well.

### 4.4 Outlook and Conclusion

Several *I_Kur_* parameters were hardly identifiable in our experiments showing the abundance of local minima of this optimization problem and the insensitivity to these parameters with respect the voltage protocol being used. One reason for this is that the voltage protocol did not cover enough of the characteristics of the channel. In particular, the second voltage step to −110 mV did not elicit a significant current. The challenging nature of the *I_Kur_* parameter estimation problem was also shown by the fact that TRR did not converge to a solution in many cases. Moreover, the rather large relative errors in Figure 8 can be due to dependencies between parameters and model sloppiness (Tøndel et al., 2014).

In this study, the ODEs underlying the ion current formulations were solved analytically as the transmembrane voltage *V_m_* was a piecewise constant function during the applied voltage protocol. In general, complex optimization algorithms using a population of particles can be computationally very expensive as the cost function needs to be evaluated numerous times. Therefore, the analytical solution facilitated the use of these algorithms since the time spent for function evaluations was drastically reduced by a factor of ≈1,000 compared to the numerical approximation of the ODEs (Wilhelms et al., 2012). The importance of a streamlined cost function implementation is highlighted by the fact that <5% of the total computation time was spent in algorithm-specific functions, whereas over 95% was spent for cost function evaluation. More complex voltage protocols can improve parameter identifiability [see, e.g., Fink and Noble (2009) and Clerx et al. (2015)] on the expense of additional computational effort. Non-piecewise constant protocols requiring numerical approximation of the gating ODEs were also suggested and can provide additional insight (Mirams et al., 2015). If the additional information contained in the input data would yield better parameter identifiability and possibly even better convergence, outbalancing the additional computational effort for a single cost function evaluation has to be assessed for each problem individually.

The presented method can potentially be enhanced by altering the cost function. Highly dynamic phases following a voltage step could be assigned higher weights. The same holds for step voltages that are considered physiologically more relevant than others. Moreover, current traces with bad signal-to-noise ratio could be erased. Besides using the sum of squared errors as the cost function as done in this work, *a priori* knowledge about the signals could be used to fit, e.g., the coefficients of mono- or bi-exponential functions. The aim of future work facilitated by the current study is to investigate the impact of drugs or mutations on different levels of integration, thus different simulation scales. The integration of measurement data occurs at the ion channel level and the simulations start at the single cell level going up to tissue simulations and even to the body surface electrocardiogram (Dössel et al., 2012). Our group and others have demonstrated how computational modeling can be used to evaluate effects on higher levels of integration by incorporating altered ion current parameterizations in whole-cell models (Benson et al., 2011; Hancox et al., 2014; Loewe et al., 2014b).

In this study, we proposed a novel hybrid strategy for parameter estimation of ion current formulations based on measurement data composed of population-based PSO and gradient-based TRR optimization. We showed that this approach yields very accurate and reliable estimates, thus providing an important tool to leverage high-throughput patch clamp systems by integrating the data into computational models in a single run. This allows to assess the complex, non-linear, and often non-intuitive effects of changes in the biophysical systems of interest.

## Author Contributions

AL, MW, MK, OD, and GS concepted the study. AL, MW, and JS implemented the algorithms and performed the *in silico* experiments. FF, DT, and ES performed the wet-lab experiments. AL, MW, and GS analyzed and interpreted the data. AL and MW drafted the manuscript. JS, MK, FF, DT, ES, OD, and GS revised the manuscript critically. All authors approved the final version of the manuscript.

## Conflict of Interest Statement

The authors declare that the research was conducted in the absence of any commercial or financial relationships that could be construed as a potential conflict of interest.
