# Supplementary material for: Parameter Estimation of Ion Current Formulations Requires Hybrid Optimization Approach to Be Both Accurate and Reliable
Source: Front Bioeng Biotechnol. 2016 Jan 13;3:209. doi: 10.3389/fbioe.2015.00209 (PMC4710757; doi:10.3389/fbioe.2015.00209)
Supplement: Supplementary file 1 [file data_sheet_1.pdf]

Supplementary Material: Parameter estimation of ion current formulations requires hybrid optimization approach to be both accurate and reliable

Axel Loewe [1,*], Mathias Wilhelms [1], Jochen Schmid [1], Mathias J. Krause [2], Fathima Fischer [3], Dierk Thomas [3], Eberhard P. Scholz [3], Olaf Dössel [1] and Gunnar Seemann [1]

*Correspondence:
Axel Loewe
publications@ibt.kit.edu

1 SUPPLEMENTARY MATERIAL & METHODS

1.1 Measurement data

In this study, measured current traces were used besides synthetic traces to parametrize the Courtemanche et al. (1998) I_{Kr}, I_{Kur}, and I_{Ks} formulations. Here, the details of the experimental protocols are given. All currents were recorded using a *Warner OC-725A* (*Warner Instruments, Hamden, CT, USA*) amplifier, low-pass filtered at 1 to 2 kHz (-3dB, four-pole Bessel filter) and digitized at 5 to 10 kHz (*Digidata 1322A, Axon Instruments, Union City, CA, USA*). The currents recorded in different cells were normalized to their maximum value, averaged, and scaled to the average maximum value.

1.1.1 hERG measurements

Human ether-à-go-go-related gene (hERG; alternative nomenclature KCNH2) encodes the α-subunit of the Kv11.1 protein carrying the rapid delayed rectifier potassium current (I_{Kr}). Wildtype hERG channels were expressed in Xenopus oocytes after injection of 46 nl cRNA solution per oocyte. After 3 to 4 days incubated at a temperature of 16°C, double micro-electrode voltage clamp experiments were performed in n = 8 cells. The tip resistances of the microelectrodes were in the range of 1 to 5 MΩ. The voltage clamp recordings were performed at room temperature (23 to 25°C). The bathing solution consisted of 5 mM KCl, 100 mM NaCl, 1.5 mM $CaCl_2$, 2 mM $MgCl_2$, and 10 mM HEPES (pH adjusted to 7.4 with NaOH) and the pipette solution contained 3 M KCl. The applied voltage clamp protocol and the corresponding current traces are depicted in Figure 7B in the paper.

1.1.2 KCNA5 measurements

KCNA5 encodes the Kv1.5 protein carrying the ultra-rapid delayed rectifier potassium current (I_{Kur}). Wildtype KCNA5 was transfected into chinese hamster ovary (CHO) cell using *Fugene* reagent (*Promega, Madison, WI, USA*) (3 μg DNA per bowl). The CHO cells were incubated at 37°C in minimum essential medium α and an atmosphere of 95% humidified air and 5% CO_2. The medium was supplemented with 100 μg/ml streptomycin sulphate, 10% fetal bovine serum, and 100 U/ml penicillin G sodium. Resistances ranged between 38 and 98 MΩ. The bathing solution consisted of 140 mM NaCl, 5 mM KCl,

1 mM $MgCl_2$*6H_2O, 10 mM HEPES, 1.8 mM $CaCl_2$*2H_2O, and 10 mM glucose monohydrate. pH was adjusted to 7.4 using NaOH. The pipette solution contained 100 mM K aspartate, 20 mM KCl, 2 mM $MgCl_2$*6H_2O, 1 mM $CaCl_2$*2H_2O, 10 mM HEPES, 10 mM EGTA, and 2 mM Na_2ATP. pH was adjusted to 7.2 using KOH; patch clamp recordings were performed at a temperature of 37°C in n = 3 cells. The applied voltage protocol and the corresponding current traces are depicted in Figure 7D in the paper.

1.1.3 KCNQ1+KCNE1 measurements

KCNQ1 encodes the α-subunit of the Kv7.1 protein carrying the slow delayed rectifier potassium channel (I_{Ks}), KCNE1 encodes the β-subunit of Kv7.1. KCNQ1 was coexpressed with KCNE1 in Xenopus oocytes after injection of 46 nl cRNA solution per oocyte. Double micro-electrode voltage clamp experiments were performed at room temperature (20 to 25°C) 2 days after injection in n = 5 cells. The tip resistances of the microelectrodes were in the range of 1 to 5 MΩ. The bathing solution consisted of 5 mM KCl, 100 mM NaCl, 1.5 mM $CaCl_2$, 2 mM $MgCl_2$, and 10 mM HEPES (pH adjusted to 7.4 with NaOH). The current and voltage electrodes were filled with 3 M KCl solution. The applied voltage protocol and the corresponding current traces are depicted in supplementary Figure S3.

1.2 Ion current formulations

This section gives the equations for the ion currents as formulated by Courtemanche et al. (1998). The adjustable parameters are marked by red color in the equations. They are named and classified as additive or multiplicative in Tables S1-S3. Moreover, their values in the original formulation together with the corresponding wide and narrow range are given.
The intracellular potassium concentration $[K]_i$ was estimated for all currents:

$$E_K = \frac{R \cdot T}{F \cdot z_K} \ln \frac{[K]_o}{[K]_i} \tag{1}$$

1.2.1 I_{Kr}

$$I_{Kr} = g_{Kr} x_r \frac{1}{1 + \exp\left(\frac{V_m + g_{Kr1}}{g_{Kr2}}\right)} (V_m - E_K) \tag{2}$$

with x_r being the gating variable. Its steady state value $x_{r\infty}$, the two rate constants α_{x_r} and β_{x_r} and the time constant τ_{x_r} are defined as follows:

$$x_{r\infty} = \frac{1}{1 + \exp\left(\frac{V_m + x_{r,m1}}{x_{r,m2}}\right)} \tag{3}$$

$$\tau_{x_r} = \frac{1}{(\alpha_{x_r} + \beta_{x_r}) \cdot x_{r,KQ10}} \tag{4}$$

$$\alpha_{x_r} = \frac{x_{r,a1}\left(V_m + x_{r,a2}\right)}{1 - \exp\left(\frac{V_m + x_{r,a2}}{x_{r,a3}}\right)} \tag{5}$$

$$\beta_{x_r} = 7.3898 \times 10^{-5} \frac{V_m + x_{r,b1}}{\exp\left(\frac{V_m + x_{r,b1}}{x_{r,b2}}\right) - 1} \tag{6}$$

1.2.2 I_{Kur}

$$I_{Kur} = g_{Kur} u_a^3 u_i \left(V_m - E_K\right) \tag{7}$$

$$g_{Kur} = g_{Kur1} + \frac{g_{Kur2}}{1 + \exp\left(\frac{V_m + g_{Kur3}}{g_{Kur4}}\right)} \tag{8}$$

$$\alpha_{u_a} = u_{a,a1} \left[\exp\left(\frac{V_m + u_{a,a2}}{u_{a,a3}}\right) + \exp\left(\frac{V_m + u_{a,a4}}{u_{a,a5}}\right)\right]^{-1} \tag{9}$$

$$\beta_{u_a} = 0.65 \left[u_{a,b1} + \exp\left(\frac{V_m + u_{a,b2}}{u_{a,b3}}\right)\right]^{-1} \tag{10}$$

$$\tau_{u_a} = \frac{1}{\left(\alpha_{u_a} + \beta_{u_a}\right) \cdot \mathrm{u}_{a,KQ10}} \tag{11}$$

$$u_{a,\infty} = \left[1 + \exp\left(\frac{V_m + u_{a,m1}}{u_{a,m2}}\right)\right]^{-1} \tag{12}$$

$$\alpha_{u_i} = u_{i,a1} \left[u_{i,a2} + \exp\left(\frac{V_m + u_{i,a3}}{u_{a,a4}}\right)\right]^{-1} \tag{13}$$

$$\beta_{u_i} = \exp\left(\frac{V_m + u_{i,b1}}{u_{i,b2}}\right) \tag{14}$$

$$\tau_{u_i} = \frac{1}{\left(\alpha_{u_i} + \beta_{u_i}\right) \cdot \mathrm{u}_{i,KQ10}} \tag{15}$$

$$u_{i,\infty} = \left[1 + \exp\left(\frac{V_m + u_{i,m1}}{u_{i,m2}}\right)\right]^{-1} \tag{16}$$

1.2.3 I_{Ks}

$$I_{Ks} = g_{Ks} x_s^2 \left(V_m - E_K\right) \tag{17}$$

$$x_{s\infty} = \frac{1}{\sqrt{1 + \exp\left(\frac{V_m + x_{s,m1}}{x_{s,m2}}\right)}} \tag{18}$$

$$\tau_{x_s} = \frac{0.5}{(\alpha_{x_r} + \beta_{x_r}) \cdot \mathrm{x}_{s,KQ10}} \tag{19}$$

$$\alpha_{x_s} = \frac{x_{s,a1}\left(V_m + x_{s,a2}\right)}{1 - \exp\left(\frac{V_m + x_{s,a2}}{x_{s,a3}}\right)} \tag{20}$$

$$\beta_{x_s} = 3.5 \times 10^{-5} \frac{\left(V_m + x_{s,b1}\right)}{\exp\left(\frac{V_m + x_{s,b1}}{x_{s,b2}}\right) - 1} \tag{21}$$

2 SUPPLEMENTARY TABLES

Supplementary Table 1. I_{Kr} parameters: besides the parameter names and units, their classification as additive (±) or multiplicative (*), the standard value from Courtemanche et al. (1998) and the parameter ranges are given.

Parameter	Unit	Type	Standard value	Narrow range	Wide range
$x_{r,a1}$	1	*	3×10^{-4}	3×10^{-5}..3×10^{-3}	3×10^{-6}..3×10^{-2}
$x_{r,a2}$	mV	±	14.1	-45.9..74.1	-105.9..134.1
$x_{r,a3}$	mV	*	-5	-50..-0.5	-500..-0.05
$x_{r,b1}$	mV	±	3.3328	-63.3328..56.6672	-123.3328..116.6672
$x_{r,b2}$	mV	*	5.1237	0.51237..51.237	0.051237..512.370
$x_{r,KQ10}$	1	*	1.0	0.1..10.0	0.01..100.0
$x_{r,m1}$	mV	±	14.1	-45.9..74.1	-105.9..134.1
$x_{r,m2}$	mV	*	-6.5	-65.0..-0.65	-650.0..-0.065
g_{Kr1}	mV	±	15.0	-45.0..75.0	-105.0..135.0
g_{Kr2}	mV	*	22.4	2.24..224.0	0.224..2240.0
g_{Kr}	nS/pF	*	0.029411765	0.002941..0.294118	0.000294..2.941177
$[K]_i$	mM	*	138.994	13.8994..1389.94	1.38994..13899.4

Supplementary Table 2. I_{Kur} parameters: besides the parameter names and units, their classification as additive (±) or multiplicative (*), the standard value from Courtemanche et al. (1998) and the parameter ranges are given.

Parameter	Unit	Type	Standard value	Narrow range	Wide range
$u_{a,a1}$	1	*	0.65	0.065..6.5	0.0065..65.0
$u_{a,a2}$	mV	±	10.0	-50..70	-110..130
$u_{a,a3}$	mV	*	-8.5	-85..-0.85	-850.0..-0.085
$u_{a,a4}$	mV	±	-30.0	-90.0..30.0	-150.0..90.0
$u_{a,a5}$	mV	*	-59.0	-590..-5.9	-5900.0..-0.59
$u_{a,b1}$	1	±	2.5	-57.5..62.5	-117.5..122.5
$u_{a,b2}$	mV	±	82.0	22.0..142.0	-38.0..202.0
$u_{a,b3}$	mV	*	17.0	1.7..170.0	0.17..1700.0
$u_{a,m1}$	mV	±	30.3	-29.7..90.3	-89.7..150.3
$u_{a,m2}$	mV	*	-9.6	-96.0..-0.96	-960.0..-0.096
$u_{a,KQ10}$	1	*	3.0	0.3..30.0	0.03..300.0
$u_{i,a1}$	1	*	1.0	0.1..10.0	0.01..100.0
$u_{i,a2}$	1	±	21.0	-39.0..81.0	-99.0..141.0
$u_{i,a3}$	mV	±	-185.0	-245.0..-125.0	-305.0..-65.0
$u_{i,a4}$	mV	*	-28.0	-280.0..-2.8	-2800.0..-0.28
$u_{i,b1}$	mV	±	-158.0	-218.0..-98.0	-278.0..-38.0
$u_{i,b2}$	mV	±	-16.0	-160.0..-1.6	-1600.0..-0.16
$u_{i,m1}$	mV	±	-99.45	-159.45..-39.45	-219.45..20.55
$u_{i,m2}$	mV	*	27.48	2.748..274.8	0.2748..2748.0
$u_{i,KQ10}$	1	*	3.0	0.3..30.0	0.03..300.0
g_{Kur1}	nS/pF	±	0.005	-59.995..60.005	-119.995..120.005
g_{Kur2}	nS/pF	*	0.05	0.005..0.5	0.0005..5.0
g_{Kur3}	mV	±	-15.0	-75.0..45.0	-135.0..105.0
g_{Kur4}	mV	*	-13.0	-130.0..-1.3	-1300.0..-0.13
$[K]_i$	mM	*	138.994	13.8994..1389.94	1.38994..13899.4

Supplementary Table 3. I_{Ks} parameters: besides the parameter names and units, their classification as additive (±) or multiplicative (*), the standard value from Courtemanche et al. (1998) and the parameter ranges are given.

Parameter	Unit	Type	Standard value	Narrow range	Wide range
$x_{s,a1}$	1	*	4×10^{-5}	4×10^{-6}..4×10^{-4}	4×10^{-7}..4×10^{-3}
$x_{s,a2}$	mV	±	-19.9	-79.9..40.1	-139.9..100.1
$x_{s,a3}$	mV	*	-17.0	-170.0..-1.7	-1700.0..-0.17
$x_{s,b1}$	mV	±	-19.9	-79.9..40.1	-139.9..100.1
$x_{s,b2}$	mV	*	9.0	0.9..90.0	0.09..900.0
$x_{s,KQ10}$	1	*	2.0	0.2..20.0	0.02..200.0
$x_{s,m1}$	mV	±	-19.9	-79.9..40.1	-139.9..100.1
$x_{s,m2}$	mV	*	-12.7	-127.0..-1.27	-1270.0..-0.127
g_{Ks}	nS/pF	*	0.12941176	0.012941..1.294118	0.001294 12.941176
$[K]_i$	mM	*	138.994	13.8994..1389.94	1.38994..13899.4

3 SUPPLEMENTARY FIGURES

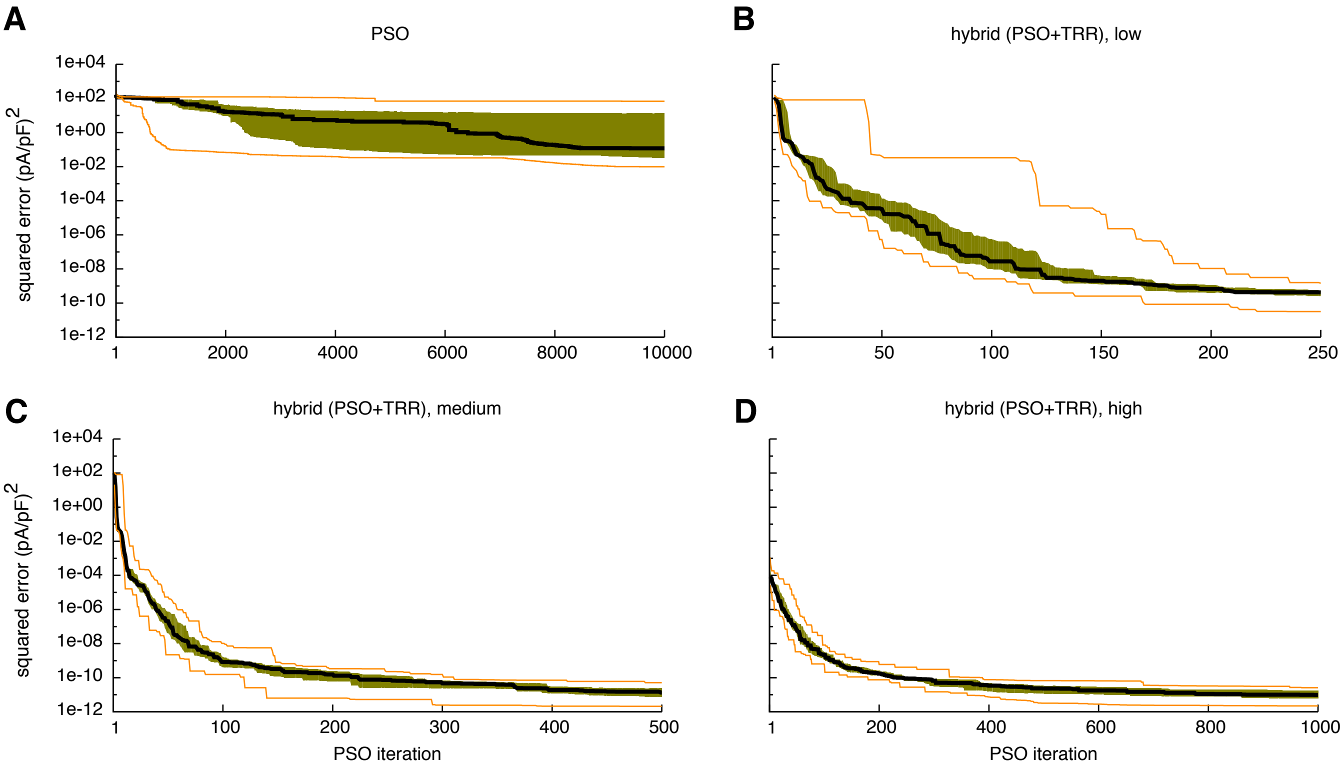

Supplementary Figure 1. Sum of squared errors convergence behavior of pure PSO (A) and *hybrid (PSO+TRR)* (B-D) for synthetic I_{Kr} data. 25 experiments were performed, the black line indicates the median, the orange lines the minimum and the maximum, the green area covers the two central quartiles. The number of particles N, the number of PSO iterations L and the number of inner TRR iterations K was increased from *"low"* (B) via *"medium"* (C) to *"high"* (D).

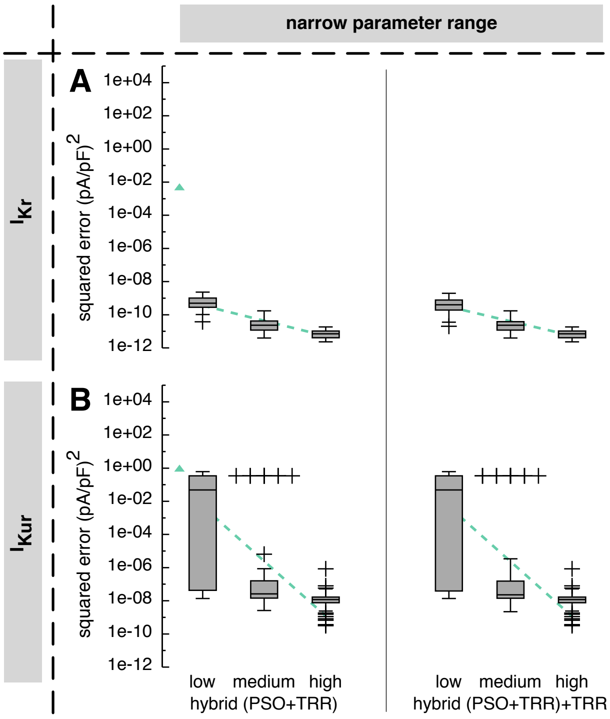

Supplementary Figure 2. Sum of squared errors achieved by *hybrid (PSO+TRR)* and *hybrid (PSO+TRR)+TRR* optimization for synthetic I_{Kr} (A) and I_{Kur} (B) data. Parameter values were restricted to the narrow ranges. The number of particles N, the number of PSO iterations L and the number of inner TRR iterations K was increased from *"low"* via *"medium"* to *"high"*. Box plots represent 25 experiments each, the dashed line indicates a linear regression of the median values in the graph coordinate system. The triangle on the left of each panel represents the median of *two-stage PSO+TRR* for N = 12,288 (compare Figure 4).

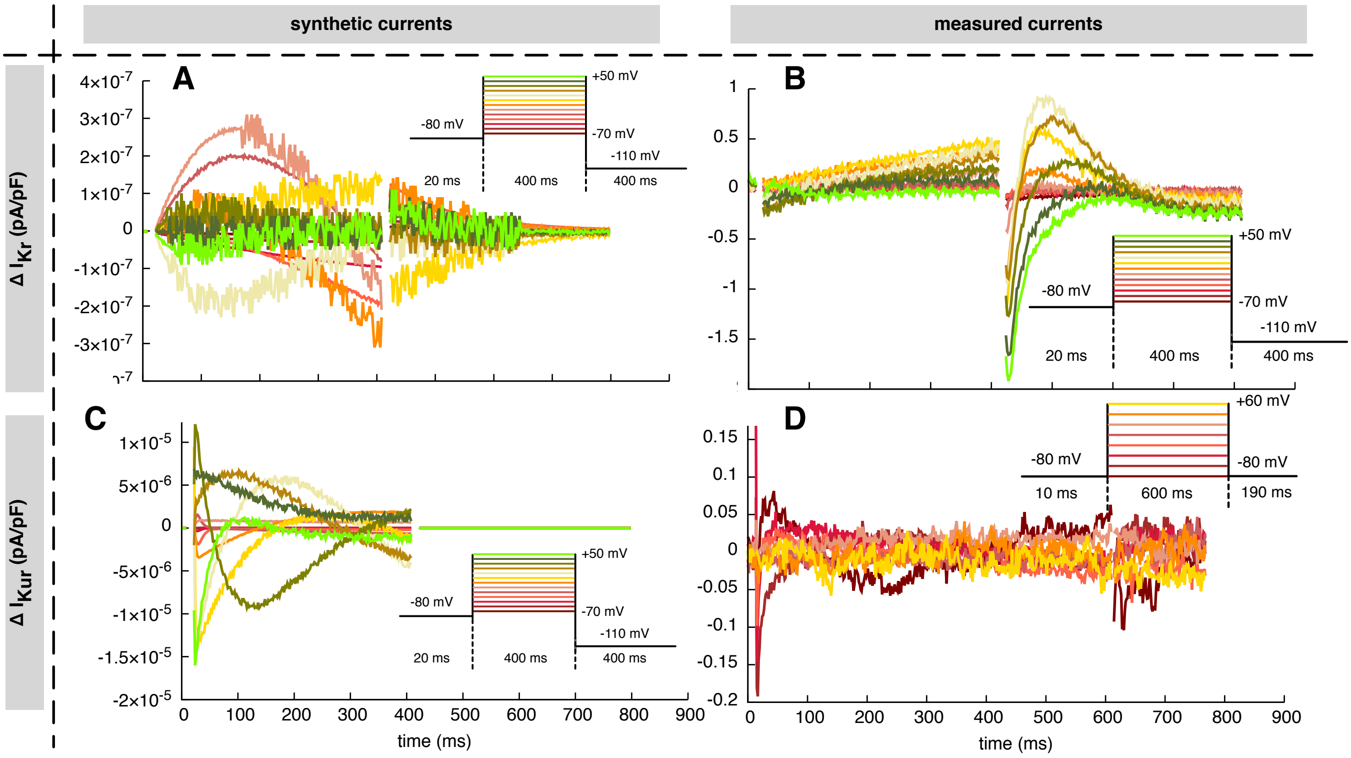

Supplementary Figure 3. Resulting difference between the measured and the simulated currents using the estimated parameters. The best fit obtained using the *"high"* setup of the *hybrid (PSO+TRR)+TRR* approach is shown. Samples directly adjacent to voltage steps were ignored in the cost function for the optimization and not plotted. (A) and (B) show I_{Kr}, (C) and (D) show I_{Kur} together with the corresponding voltage protocols.

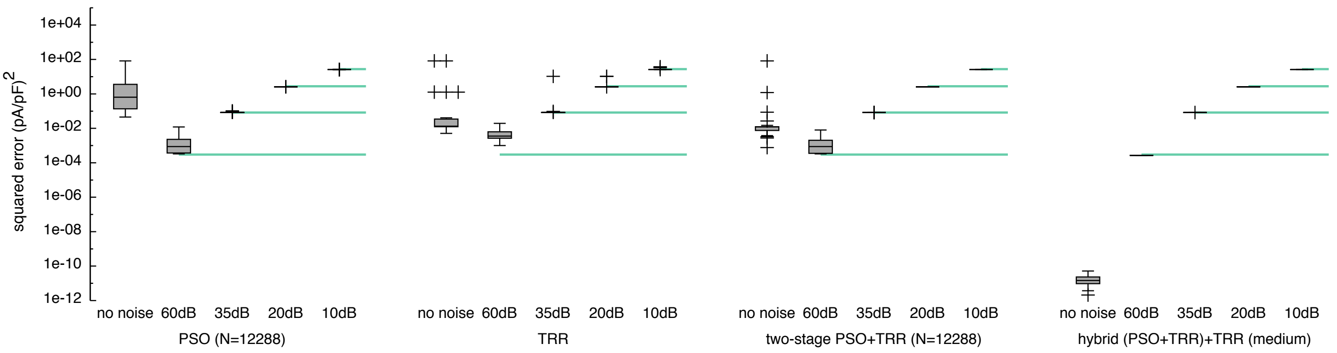

Supplementary Figure 4. Sensitivity of the sum of squared errors to noise in synthetic I_{Kr} data using pure PSO, pure TRR, *two-stage (PSO+TRR)* and *hybrid (PSO+TRR)+TRR* optimization. The squared error was measured with respect to the noisy data. Parameters were restricted to the wide ranges. Box plots represent 25 experiments. The horizontal lines indicate the sum of squared differences between the noisy input data and the ground-truth input data.

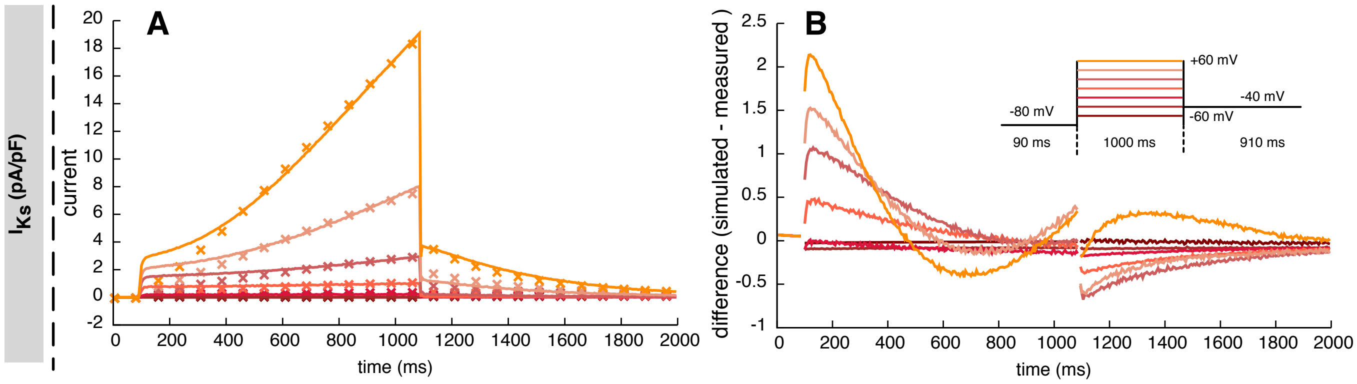

Supplementary Figure 5. Resulting I_{Ks} current using the estimated parameters together with the corresponding voltage protocol (A). Solid lines indicate measured currents used for parameter estimation. Crosses represent the best fit obtained using the *"high"* setup of the *hybrid (PSO+TRR)+TRR* approach (every 15th sample is shown). (B) shows the difference between the simulated and the measured currents. Samples directly adjacent to voltage steps were ignored in the cost function for the optimization and not plotted. Note the different scales in (A) and (B).

REFERENCES

Courtemanche, M., Ramirez, R. J., and Nattel, S. (1998). Ionic mechanisms underlying human atrial action potential properties: Insights from a mathematical model. *Am J Physiol Heart Circ Physiol* 275, H301–H321
